# Study on the Performance of Adhesive-Bolt Hybrid Connection Between GFRP Plate and Steel Plate

**DOI:** 10.3390/ma18071481

**Published:** 2025-03-26

**Authors:** Zhenchao Yang, Bin Jia, Ying Sheng, Xiao Liu, Yu Zeng

**Affiliations:** 1 School of Civil Engineer and Architecture, Southwest University of Science and Technology, Mianyang 621010, China; yzczc992@163.com (Z.Y.); shengying@swust.edu.cn (Y.S.); liuxiao0807@foxmail.com (X.L.); zengyu1125@163.com (Y.Z.); 2 Shock and Vibration of Engineering Materials and Structures Key Laboratory of Sichuan Province, Mianyang 621010, China

**Keywords:** glass fiber-reinforced polymer, damage mechanism, load–displacement curve, Hashin’s criterion, finite element analysis

## Abstract

Glass fiber-reinforced polymer (GFRP) connecting joints are difficult in the design of structural components and also critical areas prone to damage. In this study, based on the existing research, a combination of experimental and finite element analysis is used to systematically analyze the performance-influencing factors of the hybrid connection of glass fiber-reinforced composite plate and steel plate adhesive bolts under tension. By discussing the damage modes, load–displacement curves, and strain distributions at the GFRP connection joints, the influence of the connection methods and bolt quantities on the tensile properties of double-lap joints comprising GFRP plates and steel plates is revealed, and a loss evolution model for GFRP composite plates is established based on the Hashin failure criterion. The results show that the adhesive–bolted connection integrates the advantages of both adhesive bonding and bolted connections, significantly improving the tensile performance of the joint. Furthermore, the vertical arrangement of two bolts is superior to the horizontal arrangement under double-bolt connection conditions between GFRP plates and steel plates. For the several design options proposed in this study, the GFRP joints exhibit the optimal tensile properties among the four bolt arrangement schemes.

## 1. Introduction

With the rapid development of composite material technology, glass fiber-reinforced polymer (GFRP) has garnered significant attention as a composite material due to its excellent mechanical properties, outstanding corrosion resistance, and good durability [1,2,3,4]. Meanwhile, steel remains one of the primary materials in structural engineering due to its high strength, toughness, and ease of processing [5,6,7]. In the structure of a specific requirement, such as in bridge, building, and ship structures, the hybrid use of GFRP plates and steel plates can leverage the advantages of each material to achieve optimized structural design [8]. Therefore, how to efficiently and reliably connect GFRP plates and steel plates has become an important issue that needs to be addressed.

In response to this issue, adhesive–bolt hybrid connections, as an emerging connection technology [9,10], can provide a stronger and more reliable connection between different materials by combining adhesives and mechanical anchoring. Adhesive connections offer uniform stress distribution and high shear strength, while mechanical fasteners provide additional safety and tensile strength. Under tensile loading, adhesive–bolt hybrid connections must not only withstand high tensile forces but also prevent failure and debonding at the connection interface [11]. Therefore, it is of great theoretical value and practical engineering significance to investigate the mechanical properties of adhesive–bolt hybrid connections of GFRP plate and steel plate adhesive bolts when subjected to tensile loads.

Composite joints primarily utilize various connection methods, including pure adhesive connections [12,13], bolted connections [14,15], mechanical connections [12,16], and adhesive–bolt hybrid connections [17]. Despite the extensive research on these connection types, several challenges remain. The quality of the adhesive layer in adhesive bonding is difficult to control and prone to delamination [18], while bolted connections, while having strong load-bearing capacity, result in unidirectional stress and significant stress concentration. GFRP joints exhibit pseudo-ductility due to their progressive failure characteristics [19], which can mitigate some of these drawbacks. Current research has focused on the mechanical performance of pure adhesive or pure mechanical connections between composites and steel.

However, systematic research on the tensile performance of adhesive–bolt hybrid connections between GFRP plates and steel plates is relatively scarce. While some studies have investigated the principles of load distribution in hybrid connections [20] and the failure loads and modes of adhesive, bolted, and hybrid connections [21], they have not fully explored the unique mechanical behavior of double-lap hybrid connections between GFRP and steel. Gordon [22] investigated the strength and fatigue life of CFRP hybrid joints, but did not specifically focus on GFRP–steel connections. Liu [23] examined the connection performance and ductility of FRP hybrid double-lap joints, but their study did not include a detailed comparison with previous research on single-lap joints or an in-depth analysis of the failure mechanisms. Eric [24] used the hyperelastic Mooney–Rivlin potential [25,26] to finite element analysis to study joint stiffness and bonding stress distribution, but their focus was on flexible adhesives and did not specifically address GFRP–steel hybrid connections. Our study aims to bridge this gap by investigating the mechanical behavior of adhesive–bolt hybrid connections between GFRP plates and steel plates under tensile loading, using a combination of experimental and numerical simulation methods. We focus on the effects of the fastener arrangement and loading conditions on the performance of these connections, with the objective of elucidating their failure modes and mechanical characteristics. By comparing our results with previous studies on single-lap joints and other connection types, we aim to demonstrate the unique advantages and novel aspects of our proposed double-lap hybrid connection method for GFRP–steel joints. Our findings provide a scientific foundation for the design and construction of hybrid applications involving GFRP and steel structures, thereby facilitating the broader adoption of this technology in engineering practices. Notably, our research fills a critical gap in the existing literature by focusing specifically on adhesive–bolt hybrid connections between GFRP plates and steel plates, and by providing a detailed comparison with previous studies on related topics.

Researchers [19,27,28,29] have conducted preliminary studies on the load-bearing mechanisms of adhesive, bolted, and hybrid connections. The results indicate that the load-bearing mechanism of hybrid connections differs significantly from that of adhesive and bolted connections, which can enhance the load-carrying capacity of the joints. For adhesive connections, double-lap joints reduce the shear stress in the adhesive layer while weakening the peeling of the adhesive layer at the ends, thereby improving joint strength [30]. In the case of bolted connections, double-lap joints lower the peak stress and reduce stress concentration [31].

This study investigates the mechanical behavior of adhesive–bolt hybrid connections between glass fiber-reinforced polymer (GFRP) plates and steel plates under tensile loading, employing a combination of experimental and numerical simulation methods. The research examines the effects of the fastener arrangement and loading conditions on the performance of these connections, with the objective of elucidating their failure modes and mechanical characteristics. The findings aim to provide a scientific foundation for the design and construction of hybrid applications involving GFRP and steel structures, thereby facilitating the broader adoption of this technology in engineering practices. Notably, there has been a lack of specific research focused on adhesive–bolt hybrid connections. In response, this paper proposes a double-lap hybrid connection method for joints between GFRP plates and steel plates.

## 2. Experiment

In this study, hybrid joints composed of glass fiber-reinforced polymer (GFRP) plates and steel plates were designed, based on the dimensional design principles of adhesive bonding and bolted connections. The tensile performance of the orthogonally laminated GFRP–steel double-lap hybrid joints was investigated through both experimental and simulation approaches. The influence of the connection methods and the number of bolts on the tensile performance of the GFRP plates was analyzed through failure modes, load–displacement curves, and strain distribution. Static tensile tests were conducted on the adhesive bonding, bolted connections, and adhesive–bolt hybrid connections of GFRP and steel plates. Additionally, detailed analyses of the effects of different connection methods and the number of bolts, while maintaining a constant overlap area, on the tensile performance of the double-lap joints were performed through quasi-static tensile experiments.

### 2.1. Specimen Design and Fabrication

The experiment primarily investigates two conditions: the connection method and the number of bolts. The GFRP plates are made of orthogonally laminated glass fiber composite material, with each layer having a thickness of 0.15 mm. The steel plates are made of Q235 low-carbon steel, the adhesive layer uses epoxy resin, and the bolts are M6 stainless steel bolts. A double-lap connection method is employed between the GFRP plates and the steel plates. The material parameters are listed in Table 1 and Table 2. It is necessary to describe the parameters in Table 1, where E1, E2, and E3 represent the elastic moduli of the GFRP plate in three orthogonal directions, respectively; V denotes the Poisson’s ratio of the GFRP plate; G stands for the shear modulus of the GFRP material; S signifies the shear strength of the GFRP plate; Xt and Yt are the tensile strengths of the GFRP plate in the fiber direction and perpendicular to the fiber direction, respectively; Xc and Yc represent the compressive strengths in the corresponding directions.

There are currently no unified regulations for the selection of geometric parameters for hybrid connections. Based on the dimensional design of adhesive and bolted connections [32], the specimen designs for each condition are shown in Table 3. The selected overlap length for the joint specimens is 70 mm, with an adhesive layer thickness of 0.3 mm, and a plate thickness of 3 mm.

**Figure 1:**
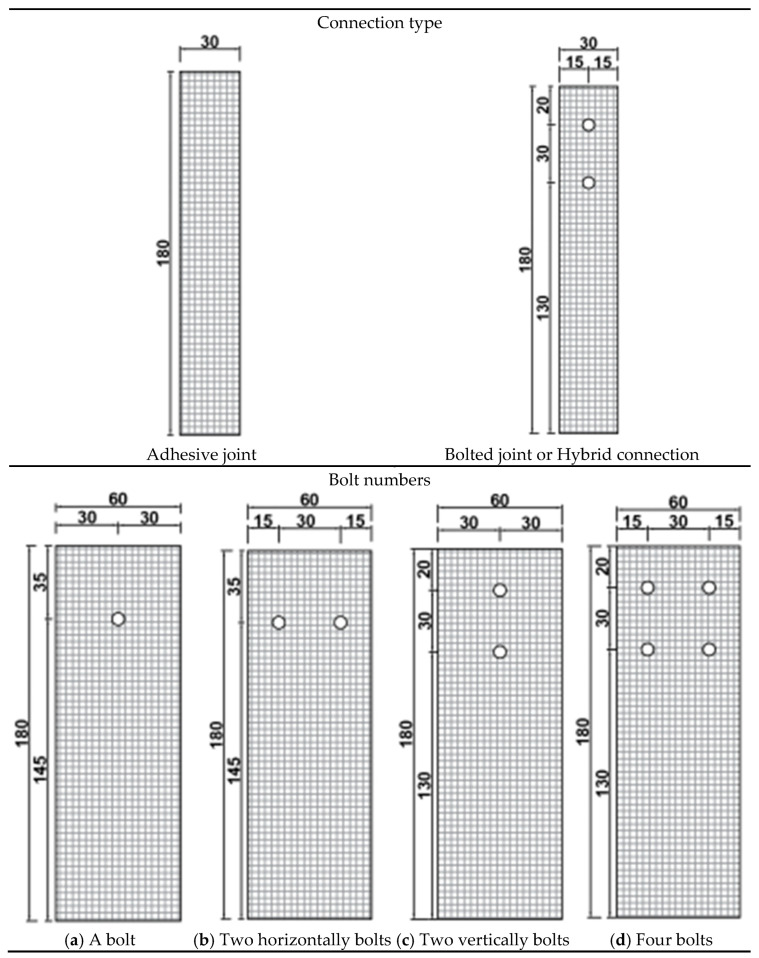
Schematic diagram of specimen design (units: mm) (note: the circular holes in the figure are all M6 screw holes).

During the fabrication process, the GFRP plates, steel plates, and reinforcement pieces were ground using a grinder, and the ground areas were treated with alcohol. The specimens were then left to stand at room temperature (25 °C) for 12 h. A 0.3 mm iron wire was placed vertically in the middle position on both sides of the GFRP plate. Epoxy resin components A and B were mixed in a ratio of 100:35, stirred for half a minute, and evenly applied to the overlapping areas on both sides of the GFRP plate. The components were assembled at the overlapping joints and a torque of 4 N·m was applied using a torque wrench. The specimens were left to cure completely before conducting the experiments. A total of 8 groups of specimens were designed, with each group consisting of 3 specimens. The design of the test specimen is shown in Figure 1.

### 2.2. Experimental Method

**Figure 2:**
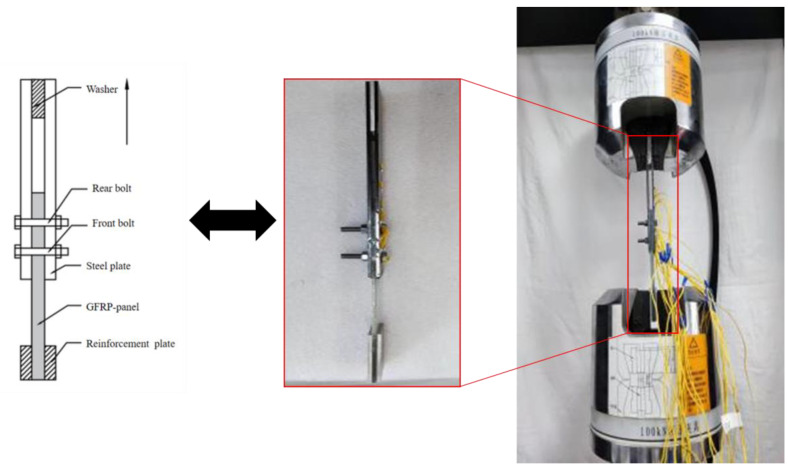
Diagram of specimen and test loading.

The experimental loading device used is the ETM-105D microcomputer-controlled electronic universal testing machine (Kason, Jinan, China), with a maximum testing force of 100 kN. The tensile tests were conducted in accordance with the standard ASTM D5961 [33], with the specimen loading configuration shown in Figure 2, where the arrows indicate the direction of load application. During the experiment, the specimens were fixed in the grips to ensure that the specimen axis was aligned with the loading direction of the testing machine. The ends of the experimental components were connected to the upper and lower grips of the universal testing machine.

During the loading process, the lower grip remained stationary while a vertical tensile load was applied through the upper grip. This experiment employed displacement-controlled loading at a speed of 2 mm/min, stopping the loading when it reached 30% of the maximum load. The testing interface recorded the maximum load and displacement from the start of the test until failure, and observations of experimental phenomena and failure modes of the joints were documented throughout the testing process.

## 3. Experimental Results and Discussion

This section will analyze the influence of joint parameters on tensile performance from the perspectives of failure modes and load–displacement curves. The failure strength of the joint is used as a criterion for measuring the tensile performance of the joint. The calculation formula for the failure strength of hybrid connection joints is as follows:

(1) $$\sigma_{b}=\frac{P}{DT}$$

where $\sigma_{b}$ is the failure strength (MPa); *P* is the ultimate load (N); *D* is the bolt diameter (mm); and *T*—GFRP plate thickness (mm).

### 3.1. The Influence of Connection Method on the Tensile Performance of GFRP–Steel Plates

**Figure 3:**
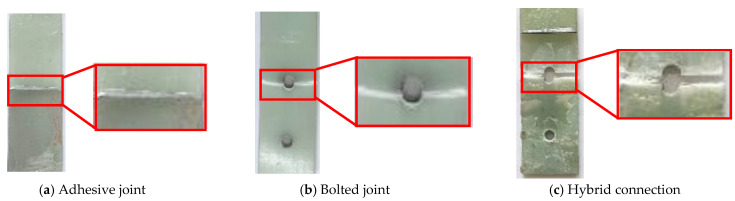
The failure modes of different connection modes.

The failure modes of each group of specimens were identical. A representative failed specimen was disassembled, as shown in Figure 3. Initially, there were no significant experimental phenomena observed in both the adhesive and hybrid connection specimens. Subsequently, sounds of adhesive layer delamination began to emerge. In the hybrid connection specimens, once the adhesive layer delaminated, the load was transferred to the bolts, leading to audible fiber breakage, with a failure mode similar to that of the bolted connections.

For the bolted connection specimens, no obvious phenomena were noted; however, a loud “pop” sound was produced at the moment of failure. Both the bolted and hybrid connections experienced tensile failure characterized by vertical fiber breakage and lateral fiber pull-out, with the hybrid connections exhibiting a greater degree of fiber pull-out compared to the bolted connections.

**Figure 4:**
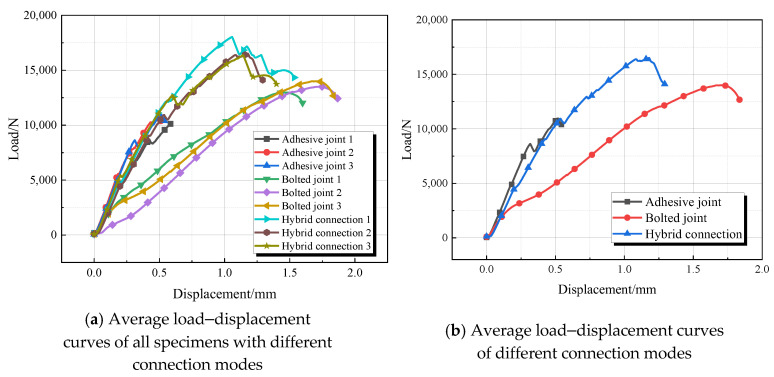
Load–displacement curve.

Due to the flowability of the epoxy resin adhesive, there was epoxy resin present on the bolts of the hybrid connection specimens, which further delayed the cracking of the adhesive layer and allowed for combined loading between the adhesive layer and the bolts. The failure mode of the adhesive and hybrid connections primarily involved mixed failure, with cracks and delamination occurring in the adhesive layer.The load-displacement curve is shown in Figure 4.

**Figure 5:**
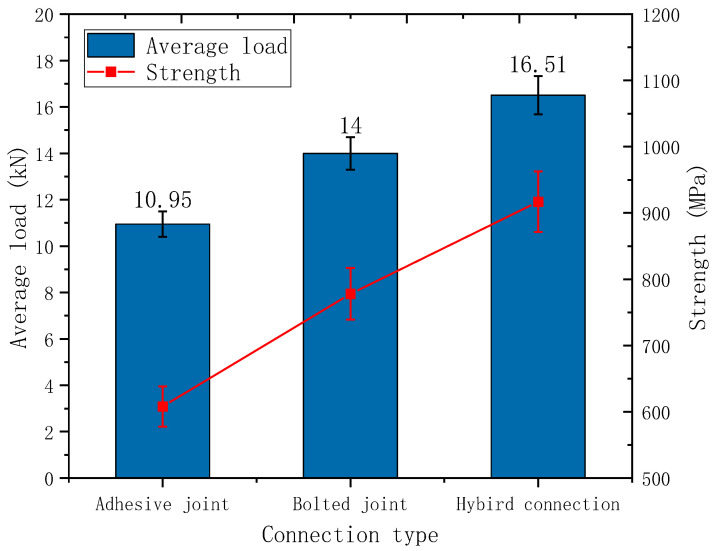
Error bars for average load and intensity (5%).

Both the bolted and hybrid connections experienced tensile failure at the end bolt holes of the GFRP plates, while delamination and micro-deformation were observed at the rear bolt holes. Both the adhesive and bolted connections exhibited brittle failure, whereas the hybrid connections demonstrated ductile failure. Figure 5 reflects the error bars for average load and strength.

### 3.2. Influence of Bolt Quantity on the Tensile Performance of GFRP–Steel Plates

**Figure 6:**
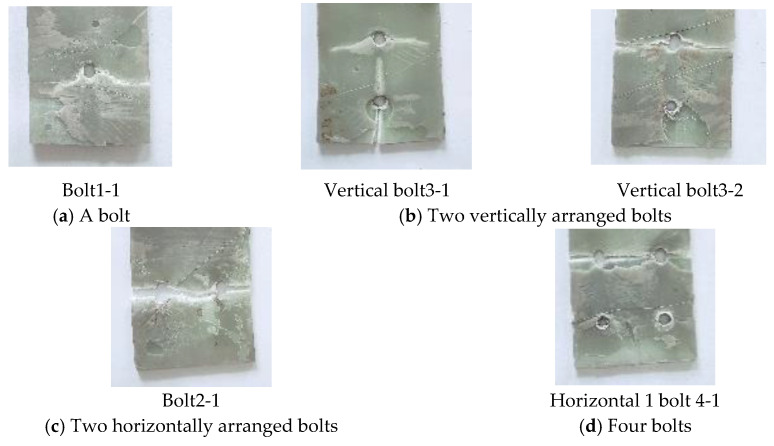
Different bolt number failure mode.

From the experimental results shown in Figure 6, it can be observed that in hybrid connections with different numbers of bolts, the adhesive layer fails first. Following the failure of the adhesive layer, continuous sounds of fiber breakage from the GFRP plate are heard until complete failure occurs. Notably, the delamination rate of the adhesive layer in vertical bolt arrangements is slower than that in horizontal bolt arrangements. Due to the difficulty in controlling the quality of the adhesive layer, different failure modes were observed. For joints with one bolt, two horizontal bolts, and four bolts, the failure mode was characterized by tensile failure of the GFRP plate at the end bolt holes. In the case of joints with two vertical bolts, two distinct failure modes were identified: for specimens Vertical bolt3-1 and Vertical bolt3-2, the failure modes were a combination of tensile and splitting failures, resulting in the bolt holes becoming elliptical in shape. The steel plate and bolts did not exhibit significant deformation.

**Figure 7:**
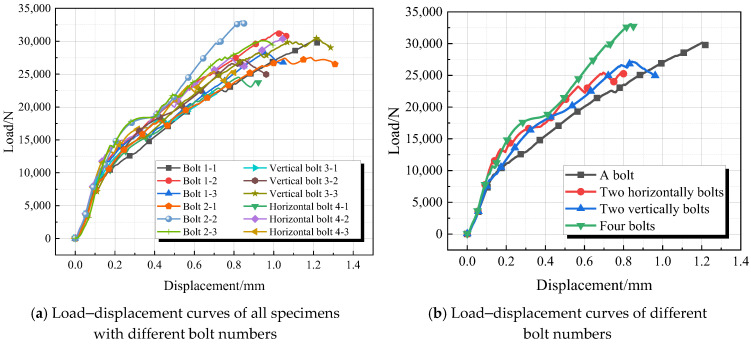
Load–displacement curve (note: in Figure 6a, Bolt1-x” denotes the adhesive bonding of a single bolt, while “Bolt2-x” represents the adhesive bonding of two bolts arranged horizontally).

The load–displacement curves for each group are shown in Figure 7. In the initial stage of the experiment, the load–displacement curves for all four groups of specimens largely overlap, indicating that the adhesive layers have not yet cracked and are bearing the same load. Subsequently, the load–displacement curves begin to diverge as the adhesive layers start to crack. Due to differences in the arrangement and number of bolts, the displacement at which the adhesive layers crack varies.

The end distances for the joints with one bolt and two horizontal bolts are the same. Once the adhesive layer begins to delaminate, at the same displacement, the load-bearing capacity of the joint with two horizontal bolts exceeds that of the joint with one bolt, while the ultimate load-bearing capacity is lower than that of the single-bolt joint. This is because the increase in strength from adding one bolt is less than the strength reduction caused by additional bolt holes in the GFRP plate. When there are two bolts, both horizontal bolts contribute simultaneously; in contrast, in a vertical arrangement, the end bolt transfers load to the upper bolt, delaying the cracking of the adhesive layer. As a result, the load-bearing capacity of the joint with two vertical bolts is slightly greater than that of the joint with two horizontal bolts.

The end distances for the joints with two vertical bolts and four bolts are identical; however, the latter exhibits greater stiffness and ductility. Although the initial delamination displacement of the adhesive layer is smaller than that of the joint with two bolts, it completely delaminates earlier than in the two-bolt joint. This indicates that increasing the number of bolts delays adhesive layer delamination. However, due to a higher number of bolts, the integrity of the adhesive layer is compromised, leading to an accelerated delamination rate once it begins. When delamination reaches the bolt locations, a larger number of bolts share the load, which delays the subsequent cracking of the adhesive layer and failure of the GFRP plate. Therefore, when there are four bolts, the strength of the hybrid connection is maximized.

From the analysis of the load–displacement curves in Figure 6 and Table 4, it can be concluded that the number of bolts significantly affects the tensile performance of hybrid connections; however, the strength does not increase linearly with an increasing number of bolts. Among them, the hybrid connection with four bolts exhibits the highest failure strength, while that with two bolts shows the lowest strength, increasing from 1417 MPa to 1837 MPa. Analyzing the reasons, firstly, in double-bolt joints, due to the presence of two bolts, the stress distribution inside the joint may become more complex. Especially when the spacing or positional relationship between the two bolts is inappropriate, stress concentration or uneven stress distribution may occur. This uneven stress distribution may lead to a decrease in the overall strength of the joint. Secondly, in double-bolt joints, the connection interface may be more complicated. If the connection interface is not properly treated, such as the presence of gaps, unevenness, contamination, or other issues, they may all affect the strength of the joint.

## 4. The Finite Element Simulation of Adhesive–Bolt Hybrid Connections Between GFRP Plates and Steel Plates

### 4.1. Failure Criteria for Composite Materials

Fiber-reinforced composite materials are composed of fibers and a matrix, allowing for laminate design and exhibiting anisotropic characteristics. The failure criteria for the fibers and the matrix differ when the composite material is subjected to loads, which means that the damage criteria for anisotropic materials differ from those for isotropic materials. When composite materials are loaded, the failure modes controlled by the matrix and fibers can occur independently or sequentially. The occurrence of failure must satisfy the stress or strain components at the time of failure, which constitutes the failure criteria for composite materials.

(2) $$\mathrm{Fiber}\;\mathrm{Tension}\;(\sigma_{11}\geq 0):\;{\left(\frac{\sigma_{11}}{X_{t}}\right)}^{2}+\frac{1}{S_{c}^{2}}\left(\sigma_{12}^{2}+\sigma_{13}^{2}\right)=1$$

(3) $$\mathrm{Fiber}\;\mathrm{Compression}\;(\sigma_{11}<\;0):-\frac{\sigma_{11}}{X_{c}}=1$$

$$\mathrm{Matrix}\;\mathrm{Tension}\;(\sigma_{22}+\sigma_{33}\geq 0):$$

(4) $${\left(\frac{\sigma_{22}+\sigma_{33}}{Y_{t}}\right)}^{2}+\frac{\sigma_{12}^{2}+\sigma_{13}^{2}+\sigma_{23}^{2}-\sigma_{22}\sigma_{33}}{S_{c}^{2}}=1$$

$$\;\mathrm{Matrix}\;\mathrm{Compression}\;(\sigma_{22}+\sigma_{33}<0):$$

(5) $$\frac{1}{Y_{c}}\left[{\left(\frac{Y_{c}}{2S_{c}}\right)}^{2}-1\right]\left(\sigma_{22}+\sigma_{33}\right)+{\left(\frac{\sigma_{22}+\sigma_{33}}{4S_{c}}\right)}^{2}+\frac{\sigma_{12}^{2}+\sigma_{13}^{2}+\sigma_{23}^{2}-\sigma_{22}\sigma_{33}}{S_{c}^{2}}=1$$

### 4.2. Damage Evolution in Composites

As the tensile load on the hybrid connection joint increases and the stress reaches the damage criterion specified by the Hashin criterion, different degrees of damage will begin to occur in the fibers and matrix within the GFRP plate. This indicates that once damage in the composite material initiates, the tensile performance of the laminate starts to change, and damage progresses immediately, leading to a degradation of material properties in the damaged region.

The damage evolution law defines the pattern of material degradation following the initiation of damage. The performance degradation models for composite materials can be categorized into two types: the Sudden Degradation Model (SDM) and the Gradual Degradation Model (GDM). The parameters that significantly influence the performance degradation of composite materials are primarily the elastic modulus and shear modulus. The stiffness degradation coefficients are given in Equations (6)–(10).

(6) $$\mathrm{Fiber}\;\mathrm{Tension}:\;E_{1}=0.07E_{1}$$

(7) $$\mathrm{Fiber}\;\mathrm{Compression}:\;E_{1}=0.14E_{1}$$

(8) $$\mathrm{Matrix}\;\mathrm{Tension}:\;E_{2}=0.2E_{2},G_{12}=0.2G_{12},\;G_{23}=0.2G_{23}$$

(9) $$\mathrm{Matrix}\;\mathrm{Compression}:{\;E}_{2}=0.4E_{2},G_{12}=0.4G_{12},\;G_{23}=0.4G_{23}$$

(10) $$\mathrm{Shear}\;\mathrm{between}\;\mathrm{Fiber}\;\mathrm{and}\;\mathrm{Matrix}:\;\mu =0,\;G_{12}=0$$

### 4.3. Model Establishment

In ANSYS Mechanical 19.2, there are four methods for modeling bolts: (1) using bonded contact at the interface, (2) replacing the bolt with beam elements based on remote point technology, (3) using a simplified solid cylinder to represent the bolt while ignoring the threads, and (4) creating a solid model of the bolt that includes threads. Different modeling approaches are required to address various problems, and the choice of modeling method can significantly impact the results of finite element analysis [34,35,36]. To better reflect real-world conditions, the third modeling method is adopted, which simplifies the bolt to a cylindrical shape without threads. The bolt head and nut are also simplified to cylindrical shapes, resulting in a solid element model.

**Figure 8:**
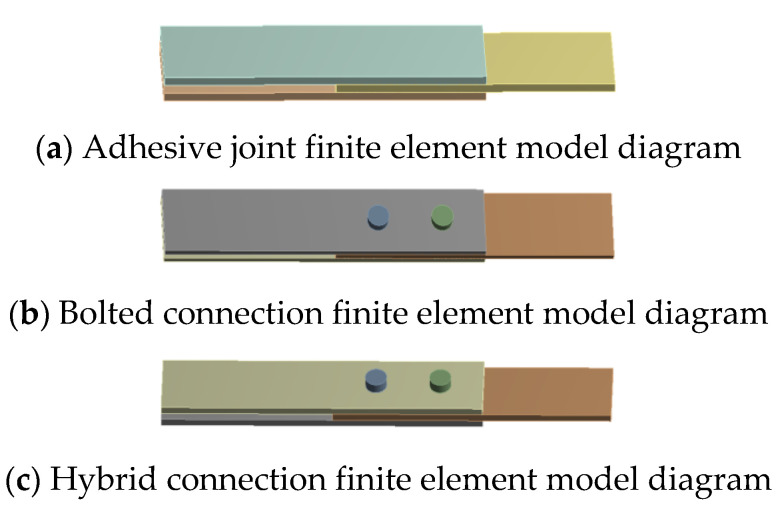
Curve of load displacement. Different connection mode model diagram.

The modeling of the GFRP plate is performed using composite shell elements to generate a solid model after layering. The steel plate is modeled using solid elements, while the adhesive layer is represented by bilinear cohesive elements. Bonded contact is applied in the contact regions. After assembling the GFRP plate, steel plate, and bolts, surface-to-surface contact is defined, and loads along with pre-tension forces are applied for analysis. The geometric parameters of the GFRP plate, steel plate, and bolts are consistent with the experimental setup, while the material parameters are provided in Table 1 and Table 2, and the geometric parameters are listed in Table 3. Models for different connection methods are illustrated in Figure 8.

### 4.4. Model Validation

In the finite element analysis (FEA) of the joints between GFRP (glass fiber-reinforced polymer) plates and steel plates presented in this paper, material nonlinearity, geometric nonlinearity, and contact nonlinearity all coexist. To better align with reality, the friction coefficient is set to 0.15 based on typical FEA settings for mechanical connections in composite materials. Due to the higher stiffness of metallic materials compared to composites, in the contact between bolts and both steel plates and GFRP plates, the bolts are designated as the master surfaces, while the steel plates and GFRP plates are designated as slave surfaces. For the contact between the steel plate and GFRP plate, the steel plate is the master surface, and the GFRP plate is the slave surface. The augmented Lagrangian contact algorithm is uniformly adopted for all contacts.

The joint model employs a mapping method for mesh generation, with a maximum mesh size of 1.5 mm. To facilitate mesh refinement in the lap joint area and around the bolts, the connecting plates and bolts are segmented, ensuring that the number of bolt mesh elements matches the number of mesh elements in the bolt holes. Both the bolt holes and the bolt circumferences are divided into 80 segments, with a bolt mesh size of 0.3 mm. To facilitate convergence in the FEA, the GFRP plate is designated as the loading end and subjected to a displacement load, while the steel plate end is constrained with fixed boundary conditions. In the first two load steps, the displacement is set to zero. The application sequence is as follows: first, the fixed constraints are applied; second, the displacement load is applied; and finally, the bolt preload is applied.

**Figure 9:**
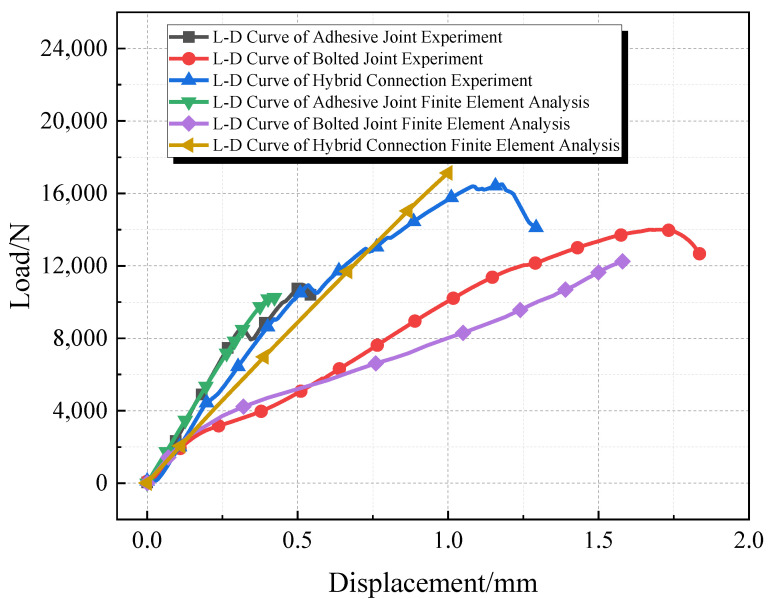
The comparison chart of load–displacement curves between experiments and finite element analysis.

Through the establishment of the finite element models for adhesive bonding, bolted connections, and hybrid joints, Finite Element Analysis (FEA) was first conducted for the adhesive and bolted connections. The results of the finite element calculations were compared with the experimental results. The ultimate load capacities for the adhesive and bolted joint experiments were 10.95 kN and 14 kN, respectively, while the ultimate load capacities from finite element analysis were 10.24 kN and 12.25 kN, resulting in errors of 6.5% and 12.5%, respectively. For the hybrid joint, the experimental and finite element analysis ultimate load capacities were 16.51 kN and 17.13 kN, with an error of 3.8%. The load–displacement curves from the experiments and finite element analyses are shown in Figure 9.

Regarding the errors arising from the experiments and simulations, we have analyzed potential sources of discrepancies, including incomplete adhesion between specimens and fixtures, material defects present in samples, and misalignment during tensile tests. Each of these factors could introduce variations that affect the mechanical properties measured in our experiments.

Incomplete Adhesion: Adhesion issues between specimens and clamping devices may lead to stress concentrations or slipping, altering the stress–strain behavior and ultimately impacting the results. To mitigate this issue, we ensured that specimens were properly prepared and clamped, acknowledging that minor imperfections might still occur.

Material Defects: Defects such as inclusions, pores, or cracks within the material can significantly influence its mechanical response under tensile loading. Although we carefully selected specimens that appeared visually free of defects, the presence of microscopic imperfections may have contributed to the observed differences.

Misalignment in Tensile Tests: Misalignment of the specimen axis with the direction of tensile force can introduce bending moments, leading to non-uniform stress distribution and premature failure. We employed alignment techniques to minimize this effect, acknowledging that slight misalignments might have occurred, affecting the accuracy of our measurements.

In addition to these considerations, we also discussed other potential sources of error, such as instrument calibration, data acquisition rates, and environmental factors (e.g., temperature variations). To further enhance the reliability of our research findings, we plan to conduct additional experimental trials with stricter controls, potentially refining specimen preparation and testing protocols. Furthermore, we will explore advanced simulation techniques to better capture the complexities of the experimental setup and material behavior.

Our comprehensive analysis of these potential sources of error demonstrates our commitment to rigorous scientific inquiry and our dedication to improving the accuracy and reliability of our results.

From the comparison of the load–displacement curves, it is evident that there are certain discrepancies between the experimental results and those from FEA during the loading process. These discrepancies can be attributed to several unavoidable factors that influence the data in both the experimental and finite element contexts. Factors affecting the experimental results include the following: control of adhesive layer quality during specimen fabrication, internal defects within the GFRP plate, and minor delaminations at the edges of the cut GFRP plates and around the bolt holes, as well as dimensional inaccuracies of the specimens. Additionally, the precision of the experimental instruments and equipment can introduce errors in the experimental data.

In finite element analysis, errors may arise from factors such as simplifications made during bolt modeling and discrepancies between selected friction coefficients and actual frictional forces. The presence of errors is inevitable; however, the observed discrepancies between the experimental and finite element analysis results fall within acceptable limits. Therefore, the establishment of the finite element model is deemed reliable.

### 4.5. The Finite Element Result and Analysis

#### 4.5.1. Connection Methods

**Figure 10:**
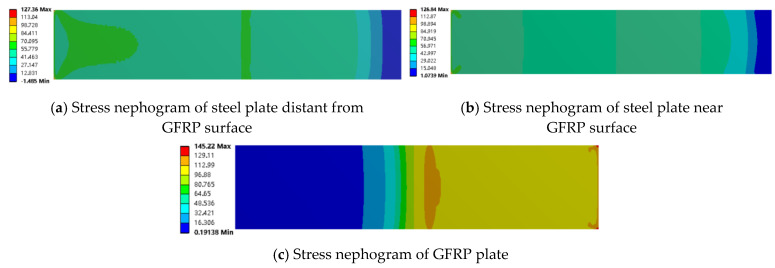
Stress nephogram of bonding along the tensile direction (x axis).

The maximum load for the adhesive joint is approximately 10 kN. Finite element results for the mixed and adhesive joints at a load of 10 kN were extracted and analyzed using stress contour plots (as shown in Figure 9 and Figure 10). The analysis reveals that under the same load, the stress values in the GFRP plate and steel plate are similar for both adhesive and mixed connections, with the stress in the GFRP plate consistently higher than that in the steel plate. This observation corresponds with the strain distribution observed in the experiments.

**Figure 11:**
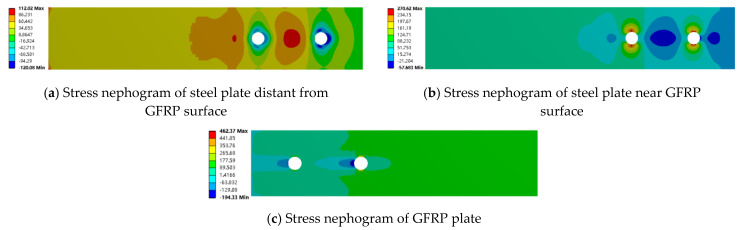
Stress nephogram of hybrid connection along tensile direction (x axis).

From Figure 10 and Figure 11, it can be seen that in both the adhesive and mixed connections, the stress in the steel plate near the GFRP plate surface and in the non-overlapping region away from the GFRP plate is nearly identical. However, there is a significant difference in stress within the overlapping region of the mixed connection, particularly around the bolt holes, where stress concentration is evident. The tensile stress limit for the GFRP plate is 442 MPa, and the compressive stress limit is 337 MPa. In the adhesive connection, the maximum stress in the GFRP plate reaches 145.22 MPa, indicating that failure of the component occurs before the GFRP plate itself fails, resulting in lower load-bearing capacity. In the mixed connection, the presence of bolt holes compromises the integrity of the GFRP plate, with maximum stress occurring at the bolt hole, reaching 446.27 MPa, indicating that failure initiates at this location.

The maximum load for the bolted joint is approximately 12 kN. Therefore, finite element results for the mixed and bolted connections at a load of about 12 kN were extracted and analyzed using stress contour plots. From Figure 8, it can be observed that the stress in the non-overlapping region is relatively low, while higher stress is primarily concentrated in the overlapping region. Under the same external load, the stress values in the GFRP plate and steel plate for both the bolted and mixed connections are similar, with the stress in the GFRP plate consistently greater than that in the steel plate. This indicates that the presence of the adhesive layer mainly affects the stress distribution in the overlapping region, while its impact on the stress concentration in the steel plate is minimal. Conversely, the adhesive layer significantly influences the stress concentration in the GFRP plate, resulting in much higher stress values in the GFRP plate compared to those in the steel plate.

From Figure 10 and Figure 11, it can be observed that in both the bolted and mixed connections, the stress concentration in the steel plate near the GFRP plate surface is significantly greater than that in the steel plate away from the GFRP plate surface. This is due not only to the compression exerted by the bolts on the steel plate near the surface but also to the frictional forces between the steel plate and the GFRP plate. In the mixed connection, there is also a peeling force from the adhesive layer. The adhesive layer hinders joint failure, resulting in a greater variation in stress in the steel plate located away from the GFRP plate surface in the bolted connection compared to that near the surface, while the variation in stress is smaller in the mixed connection.

**Figure 12:**
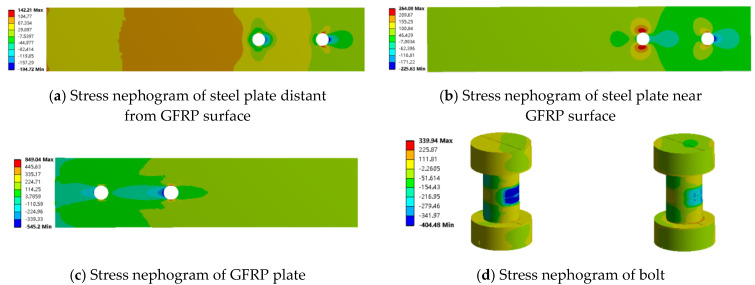
Stress nephogram of bolted connection along tensile direction (x axis).

In the bolted connection, stress decreases from the overlapping region to the non-overlapping region, with higher stress values closer to the surface and minimal variation. This trend aligns with that observed in the mixed connection. As shown in Figure 12, it was noted that on both sides of the bolt holes perpendicular to the load direction near the GFRP plate surface, tensile forces are acting, resulting in positive stress values; conversely, compressive forces act on the bolt holes aligned with the load direction. A key difference is that in the mixed connection, compressive forces act on both sides of the bolt hole located below the steel plate away from the GFRP plate surface, while in the bolted connection, this position experiences compressive forces as well. This illustrates the influence of the adhesive layer on the stress distribution around the bolt holes on either side of the steel plate.

Additionally, it was observed that the stress concentration at the bolt hole behind the GFRP plate is significantly greater than that at the front-end bolt hole. The stress concentration at the front-end bolt holes in both the mixed and bolted connections is nearly identical. The failure range of the GFRP plate is larger than that in the mixed connection, with both failure regions located at points of contact with the GFRP plate. Under the same loading conditions, the stress concentration of the hybrid connection is lower compared to the bolted connection, which further confirms that the adhesive layer has a significant effect on the bolt stress state.

From this analysis, it can be concluded that in the case of adhesive bonding, although the presence of bolts may accelerate the failure of the GFRP plate, it enhances the tensile performance of the joint, allowing the GFRP plate to function effectively. In mixed connections, the adhesive layer does not have a significant impact on the stress distribution in the non-overlapping region, which reduces the extent of damage to the GFRP plate. However, the presence of bolts exacerbates the stress concentration, allowing the GFRP plate to perform effectively and improving the strength of the joint. Therefore, the mixed connection method provides better tensile performance for the connection between the GFRP plate and steel plate.

#### 4.5.2. Number of Bolts

**Figure 13:**
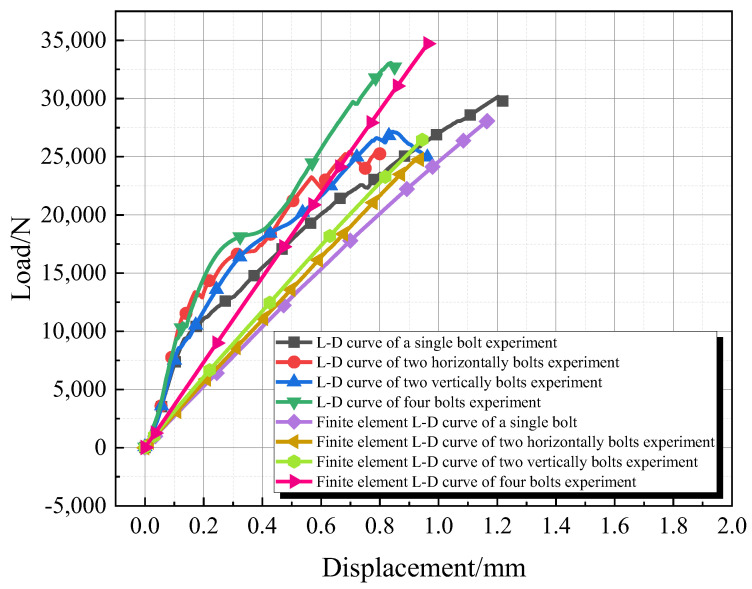
The comparison of load–displacement curves between test and finite element analysis.

The comparison of load–displacement curves from the experiments and finite element analysis is shown in the following figure. From Figure 13, it can be observed that the discrepancies between the experimental results and finite element analysis are within an acceptable range.

**Figure 14:**
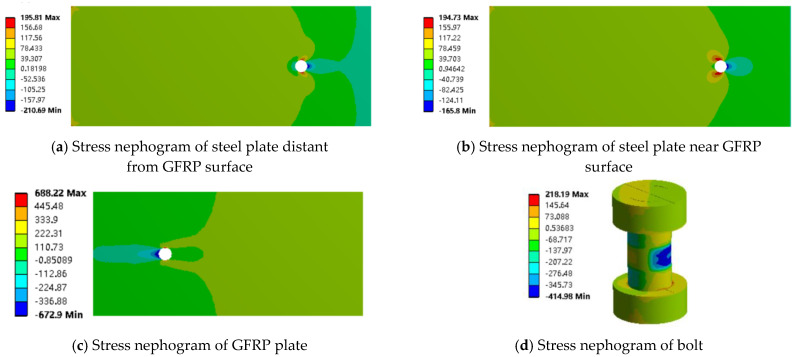
Stress nebulae of a bolted joint along the tensile direction (x axis).

In the experiment examining the effect of bolt quantity on the tensile performance of mixed joints, the ultimate load capacity of a joint with one bolt is approximately 30 kN, while the ultimate load capacity of a joint with two horizontally arranged bolts is approximately 25 kN. Therefore, finite element analysis was conducted at a load of about 25 kN for both cases, and the stress contour plots were analyzed. It was found that the stress distribution for the two horizontally arranged bolts is symmetrical. The finite element analysis results for one bolt in the load direction are presented in Figure 14.

**Figure 15:**
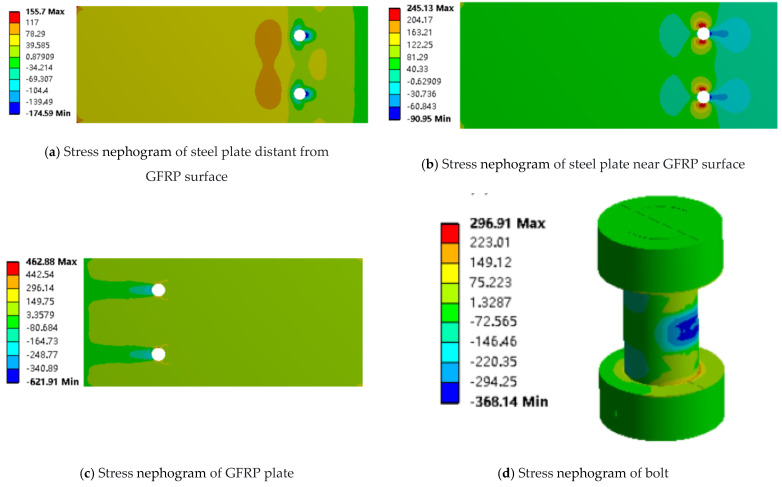
Stress nebulae of two horizontal bolt joints along the tensile direction (x axis).

**Figure 16:**
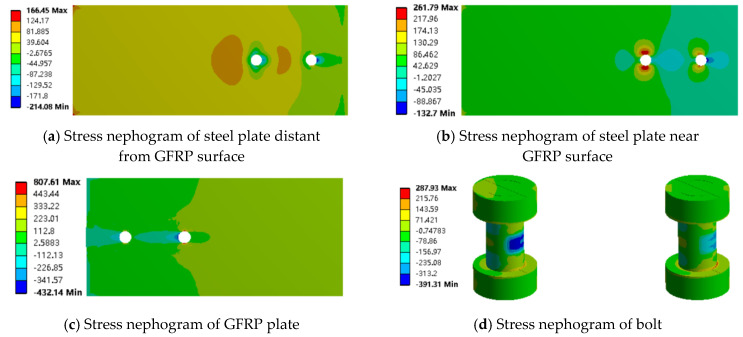
Stress nebulae of two vertical row bolt joints along the tensile direction (x axis).

In the experimental analysis of joints with different numbers of bolts, the ultimate load capacity of the joint with two horizontally arranged bolts is approximately 25 kN, while the ultimate load capacity of the joint with two vertically arranged bolts is approximately 27 kN. Therefore, the stress contour plots along the load direction at a load of about 25 kN were analyzed. It was found that in the joint with two horizontally arranged bolts, the stress distribution of the two bolts is symmetrical; thus, only the finite element results for one bolt in the load direction are presented, as shown in Figure 15. The finite element analysis results for the joint with two vertically arranged bolts are shown in Figure 16.

The different arrangement of the bolts mainly affects the stress state in the lap joint region. Specifically, pressure is prevalent at the holes of the steel plate away from the GFRP plate surface, and the stress concentration at the front holes is higher than that at the rear holes of the bolts in the vertically bolted joints. In addition, both sides of the screw holes close to the GFRP plate face are subjected to tensile force, and the stress concentration at the rear bolt holes of the plate is more significant in the vertical bolted joints, which leads to the increase in the degree of damage and failure of the GFRP plate. Through the above analysis, it can be seen that although the arrangement of the vertical row of bolts reduces the load borne by the bolts to a certain extent and improves the strength of the joints, it also exacerbates the problem of stress concentration in the GFRP plate.

**Figure 17:**
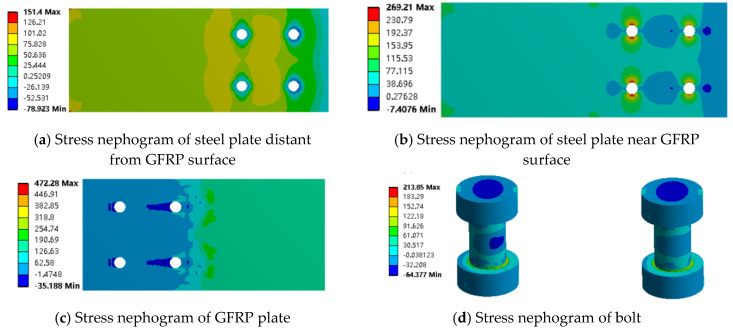
Stress nephogram of four bolted joints along the tensile direction (x axis).

In the analysis of the number of bolts, the ultimate load capacity of the joint with two vertically arranged bolts is approximately 27 kN, while the ultimate load capacity of the joint with four bolts is approximately 33 kN. Therefore, the stress contour plots at a load of about 27 kN were analyzed. It was found that in the joint with four bolts, the stress distribution of the bolts along the load direction is symmetrical; thus, only the finite element results for the two bolts in the load direction are presented, as shown in Figure 17.

The increase in the number of bolts does not have a significant effect on the stress distribution of the steel plate, but it has a greater effect on the GFRP plate and the bolts, which is reflected in the reduction in the stress concentration of the bolts. In addition, increasing the number of bolts can reduce the load borne by the GFRP plate, which in turn reduces the degree of damage and improves the overall strength of the joint, making the load distribution between the bolts more uniform.

## 5. Conclusions

This study focuses on the tensile properties of GFRP plate–steel plate lap joints, and systematically investigates the specific effects of different connection methods and number of bolts on the tensile properties of hybrid GFRP plate–steel plate joints through experiments and finite element analysis. The following conclusions were drawn:

1. The advantages of adhesive–bolt hybrid connections: The adhesive–bolt hybrid connection method combines the benefits of adhesive bonding and bolted connections. The adhesive layer bears a significant portion of the load, primarily influencing the stress distribution in the overlap region of the GFRP plate. The presence of bolts reduces the delamination rate of the adhesive layer, allowing for concurrent loading of both the adhesive and bolts, which diminishes the damage to the GFRP plate, delays joint failure, and enhances the connection performance. However, this configuration also exacerbates the stress concentration compared to pure adhesive or bolted connections.
2. Impact of bolt quantity on joint strength: For a constant overlap area, the number of bolts significantly affects the strength of the joint by influencing the load distribution among the GFRP plate, steel plate, and bolts in the overlap region. The joint strength does not increase linearly with the number of bolts; the hybrid joint with four bolts exhibits the highest strength while also reducing the stress concentration. Furthermore, a single-bolt arrangement is superior to a double-bolt configuration. When bolts are aligned along the load direction, the joint demonstrates improved tensile performance. However, when designing hybrid joints with a high number of bolts, the quality of the adhesive layer may be compromised, which in turn makes the failure mode predominantly tensile damage.
3. In practical engineering applications, several factors must be considered regarding the connection between GFRP plates and steel plates, including the GFRP plate thickness, bolt material, friction coefficient, and preload. Additionally, the quality of the adhesive layer can be affected by human and environmental factors, leading to variations in thickness, voids, and bubbles, which can compromise joint integrity. Moreover, based on the conclusions drawn from this study, genetic algorithms or other optimization techniques can be employed to optimize the shape and dimensions of the joints to achieve optimal structural configurations.

## Figures and Tables

**Table 1:** GFRP plate material parameters.

$E_{1}$ **/MPa**	23,100	$V_{12}$	0.15	$G_{12}$ **/MPa**	1600
$E_{2}$ **/MPa**	23,100	$V_{13}$	0.25	$G_{13}$ **/MPa**	1800
$E_{3}$ **/MPa**	6870	$V_{23}$	0.25	$G_{23}$ **/MPa**	1800
$S_{12}$ **/MPa**	40	$X_{t}$ **/MPa**	442	$X_{c}$ **/MPa**	337
$S_{13}$ **/MPa**	45	$Y_{t}$ **/MPa**	442	$Y_{c}$ **/MPa**	337
$S_{23}$ **/MPa**	45	$Z_{t}$ **/MPa**		$Z_{c}$ **/MPa**	

**Table 2:** Steel plate and bolt material parameter.

Composition	Elastic Modulus/GPa	Poisson’s Ratio/MPa	Yield Strength/MPa	Shear Modulus/MPa
Steel plate	210	0.3	234	1450
Bolt	193	0.27	207	692

**Table 3:** Load–displacement and strength of specimens with different connection modes.

Connection Type	Adhesive Joint	Bolted Joint	Hybrid Connection
Average load/kN	10.95	14	16.51
Displacement/mm	0.52	1.71	1.18
Strength/MPa	608	778	917

**Table 4:** Load–displacement and strength of specimens with different bolt numbers.

Bolt Numbers	A Bolt	Two Vertically Arranged Bolts	Two Horizontally Arranged Bolts	Four Bolts
Average load/kN	30.16	25.51	27.16	33.07
Displacement/mm	0.52	1.71	0.84	0.84
Strength/MPa	1676	1417	1509	1837

## Data Availability

The datasets presented in this article are not readily available because the data are part of an ongoing study. Requests to access the datasets should be directed to the corresponding author.
